# Scanner-based real-time three-dimensional brain + body slice-to-volume reconstruction for T2-weighted 0.55-T low-field fetal magnetic resonance imaging

**DOI:** 10.1007/s00247-025-06165-x

**Published:** 2025-01-24

**Authors:** Alena Uus, Sara Neves Silva, Jordina Aviles Verdera, Kelly Payette, Megan Hall, Kathleen Colford, Aysha Luis, Helena Sousa, Zihan Ning, Thomas Roberts, Sarah McElroy, Maria Deprez, Joseph Hajnal, Mary Rutherford, Lisa Story, Jana Hutter

**Affiliations:** 1 Research Department of Early Life Imaging, School of Biomedical Engineering and Imaging Sciences, King’s College London, St Thomas’ Hospital, London, SE1 7EH UK; 2 Clinical Scientific Computing, Guy’s and St Thomas’ NHS Foundation Trust, London, UK; 3 MR Research Collaborations, Siemens (United Kingdom), Camberley, UK; 4 Smart Imaging Lab, Radiological Institute, Universitätsklinikum Erlangen, Erlangen, Germany; 5 Research Department of Imaging Physics and Engineering, School of Biomedical Engineering and Imaging Sciences, King’s College London, London, UK; 6 Department of Women & Children’s Health, King’s College London, London, UK

**Keywords:** Fetus, Image reconstruction, Magnetic resonance imaging, Real-time

## Abstract

**Background:**

Motion correction methods based on slice-to-volume registration (SVR) for fetal magnetic resonance imaging (MRI) allow reconstruction of three-dimensional (3-D) isotropic images of the fetal brain and body. However, all existing SVR methods are confined to research settings, which limits clinical integration. Furthermore, there have been no reported SVR solutions for low-field 0.55-T MRI.

**Objective:**

Integration of automated SVR motion correction methods directly into fetal MRI scanning process via the Gadgetron framework to enable automated T2-weighted (T2W) 3-D fetal brain and body reconstruction in the low-field 0.55-T MRI scanner within the duration of the scan.

**Materials and methods:**

A deep learning fully automated pipeline was developed for T2W 3-D rigid and deformable (D/SVR) reconstruction of the fetal brain and body of 0.55-T T2W datasets. Next, it was integrated into 0.55-T low-field MRI scanner environment via a Gadgetron workflow that enables launching of the reconstruction process directly during scanning in real-time.

**Results:**

During prospective testing on 12 cases (22–40 weeks gestational age), the fetal brain and body reconstructions were available on average 6:42 ± 3:13 min after the acquisition of the final stack and could be assessed and archived on the scanner console during the ongoing fetal MRI scan. The output image data quality was rated as good to acceptable for interpretation. The retrospective testing of the pipeline on 83 0.55-T datasets demonstrated stable reconstruction quality for low-field MRI.

**Conclusion:**

The proposed pipeline allows scanner-based prospective T2W 3-D motion correction for low-field 0.55-T fetal MRI via direct online integration into the scanner environment.

**Graphical Abstract:**

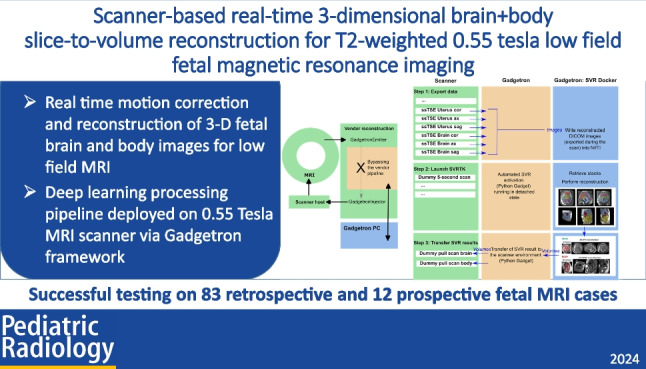

## Introduction

Fetal magnetic resonance imaging (MRI) is increasingly utilized adjunct to ultrasound for improved diagnostic accuracy [1] as well as providing a detailed characterization of normal and abnormal fetal development [2, 3].

Fast acquisition protocols such as T2-weighted (T2W) half-Fourier acquisition single-shot turbo spin echo (ssTSE) provide high in-plane two-dimensional (2-D) image quality [4]. However, unpredictable fetal motion remains the main limiting factor in both T2W 2-D in-plane artifacts and information content related to the loss of T2W three-dimensional (3-D) structural continuity.

Currently, retrospective motion correction performed in the image domain [5] has proven to be the most effective solution for structural fetal MRI. These methods are based on slice-to-volume registration (SVR) and super resolution (SR) for the reconstruction of high-resolution T2W 3-D isotropic images from multiple motion-corrupted stacks acquired under different orientations. The reconstructed volumes can be reoriented in any plane and provide true spatial information in 3-D. Originally proposed over a decade ago for the fetal brain [6–8], these methods evolved into fast deep learning automated pipelines for the whole fetus. The recent developments allow correction of nonrigid motion using deformable SVR (DSVR) for the fetal body [9, 10], deep learning masking [11], deep learning reorientation of the fetal brain [12–14] and thorax [15], and deep learning super-resolution reconstruction [10, 16, 17] that reportedly result in improved image quality and faster performance.

Yet, these methods are still primarily in the research stage. Wider integration into diagnostic practice would require a comprehensive formal evaluation of added clinical value as well as optimization of approaches for deployment directly into clinical settings. One of the major limitations of all existing solutions is that they rely on scripts executed offline on local research workstations after the fetal scan is completed (i.e., using downloaded files in neuroimaging informatics technology initiative (NIfTI) or digital imaging and communications in medicine (DICOM) formats), and do not provide a user-friendly interface or formalized data storage structures. This inherently precludes the use of T2W 3-D reconstructions in clinical radiology reporting workflow for fetal MRI.

To our knowledge, there have been no proposed SVR solutions integrated directly into the scanner environment which would allow direct export of T2W 3-D images into a picture archiving and communications system (PACS) (or similar systems) without any external processing. Several fetal MRI research works already reported automated scanner-based solutions including real-time brain tracking [18], planning of brain acquisition planes [19], and re-acquisition of low-quality slices [20]. These workflows were implemented using interaction interfaces between the scanner and external graphics processing unit (GPU) accelerated research reconstruction workstations, such as Gadgetron. Gadgetron is an open-source, modular software platform for sending and retrieving data to and from the scanner reconstruction environment and an external server. It has previously successfully facilitated the integration of several image processing pipelines into scanner environments, allowing cloud-based reconstruction of free-breathing motion-corrected cine images, quantification of myocardial perfusion, and segmentation of cardiac cavities [21–24].

Recent advancements have seen the re-emergence of low-field MRI at 0.55 T [25]. While fetal imaging is traditionally performed at 1.5 T and 3 T, 0.55 T offers solutions to several challenges associated with fetal MRI: the increased homogeneity of the magnetic field decreases magnetic susceptibility artifacts frequently found between the fetal brain and the maternal bowel and thereby also the need for specialist shimming tools as commonly used on higher field [26]. Furthermore, commercial 0.55-T scanners feature larger bore sizes, enabled by the reduced field strength requirement, significantly widening access to fetal MRI, particularly for late gestations and the growing population of pregnant women with high body mass index (BMI). However, the drawback of reduced signal-to-noise ratio (SNR) and lower resolution must be accounted for when developing low-field specific techniques.

Application of T2W 3-D reconstruction techniques could potentially improve image quality and information content of low-field datasets. Recent works [27, 28] already demonstrated the general feasibility of 0.55-T MRI for fetal brain and body imaging, as well as several examples of fetal brain SVR reconstruction and functional brain and body reconstruction [29, 30]. Yet, there has been no extensive evaluation of D/SVR performance (or dedicated solutions) to confirm the general feasibility of using T2W 3-D reconstruction at 0.55 T on a large scale.

This work integrates a T2W 3-D brain + body rigid and deformable SVR (D/SVR) reconstruction pipeline for T2W structural low-field 0.55-T fetal MRI integrated directly into the scanner environment and makes the final T2W 3-D reconstructions available during the ongoing fetal scan. It combines the previous works on automated T2W 3-D D/SVR reconstruction [15, 31] in SVR toolbox (SVRTK)^1^ optimized to 0.55-T MRI with a real-time integrated scanner workflow [18, 19] via Gadgetron.^2^ In the context of this work, real-time integration refers to the triggering of the SVR reconstruction process immediately upon the acquisition of the last T2W stack, with the retrieval of the resulting T2W 3-D images in the duration of the scan. The T2W 3-D reconstructed files are sent back to the scanner and can be directly exported to PACS.

The feasibility of the proposed automated D/SVR reconstruction pipeline is evaluated (both quantitatively and qualitatively) on 83 retrospective 0.55-T T2W datasets. Evaluation of the scanner deployment is based on real-time (in utero) testing on 12 prospective cases in terms of general operability, time, quality of the results, and operator experience. In addition to integration into the scanner environment, this is the first work on automated T2W 3-D reconstruction of T2W 0.55-T fetal datasets.

## Materials and methods

**Fig. 1:**
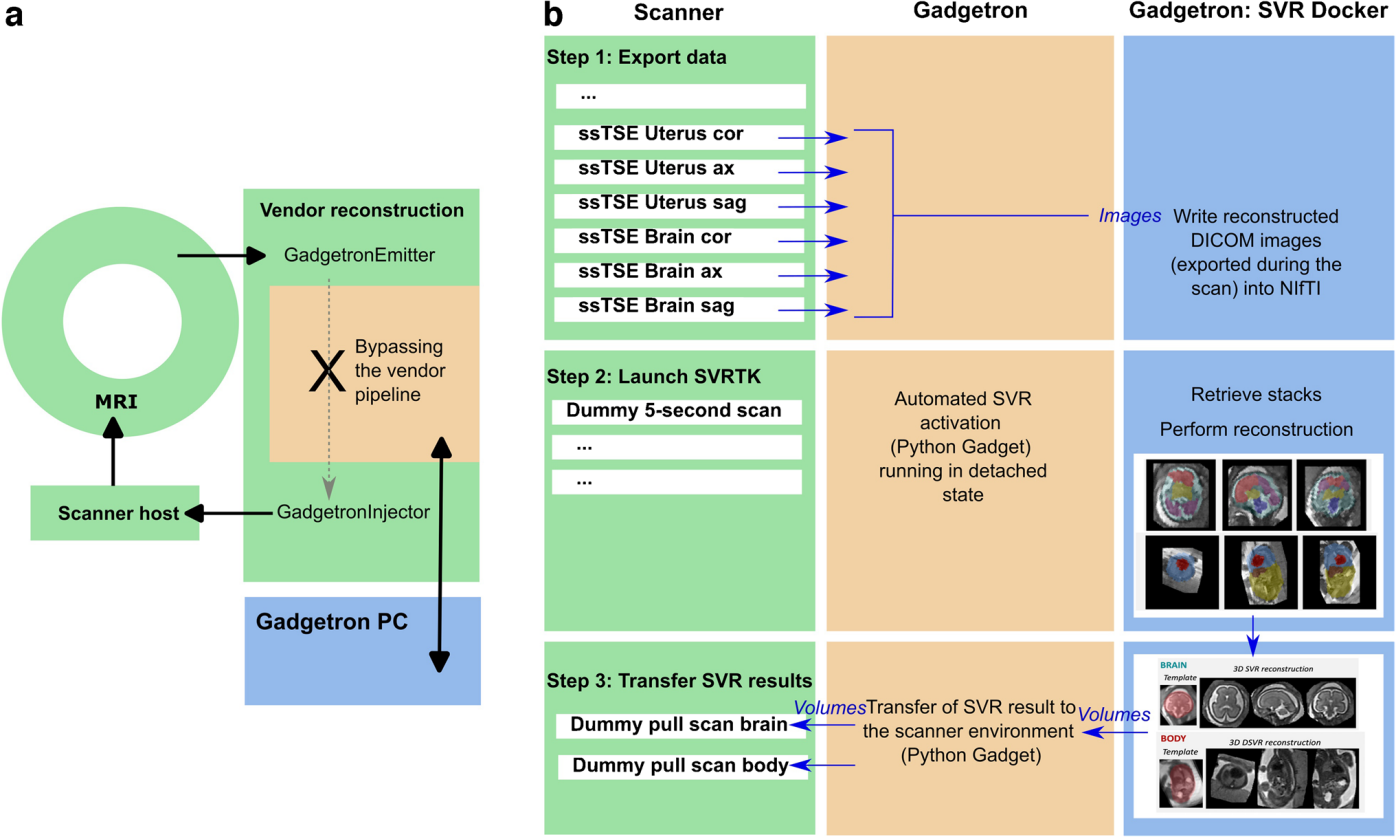
Proposed pipeline for the integration of T2W 3-dimensional brain SVR and body DSVR reconstruction into scanner environment via Gadgetron. **a** Infrastructure setup. **b** Three-step process and flow of images and volumes between the scanner, during the acquisition of the single-shot turbo spin echo scans in coronal, axial, and sagittal orientations; the Gadgetron server; and the SVR container within the Gadgetron server. *DICOM*, digital imaging and communications in medicine; *DSVR*, deformable slice-to-volume reconstruction; *MRI*, magnetic resonance imaging; *NIfTI*, neuroimaging informatics technology initiative; *PC*, personal computer; *ssTSE*, single-shot turbo spin echo; *SVR*, slice-to-volume reconstruction; *SVRTK*, slice-to-volume reconstruction toolkit

An overview of the proposed automated workflow implemented for a 0.55-T scanner is presented in Fig. 1. At first, the ssTSE images are exported to an external GPU-accelerated server (“Gadgetron server”) in real-time. Upon the collection of all ssTSE stacks, a 5-s dummy sequence is run for launching the SVR docker container (optimized for 0.55-T T2W contrast) on the Gadgetron server. Once the D/SVR results are available, 1-s dummy sequences are added to the exam card to pull the resulting T2W 3-D images to the MRI scanner and store them in PACS.

### Datasets, acquisition

The fetal MRI data used in this study were acquired at St Thomas’ Hospital, London, as part of the ethically approved MEERKAT (REC: 21/LO/0742), MiBirth (REC: 23/LO/0685), and NANO (REC: 22/YH/0210) studies. All experiments were performed in accordance with relevant guidelines and regulations. Informed written consent was obtained from all participants.

The acquisitions were performed on a contemporary clinical 0.55-T scanner (MAGNETOM Free.Max, Siemens Healthcare, Erlangen, Germany) with a six-element flexible coil (BioMatrix Contour Coil, Siemens Healthcare, Erlangen, Germany) and a nine- element spine coil built into the patient table. The structural T2W stacks were acquired using a dedicated ssTSE sequence optimized for fetal imaging at 0.55 T (low field) [27] with TR = 1,460–2,500 ms, TE = 105–106 ms, 1.4-mm in-plane resolution, and 4.5-mm slice thickness. Each dataset includes 6–12 stacks covering the whole uterus, brain, and trunk regions using standard radiological orientations. The stacks were acquired in consecutive order without time gaps or changes in the maternal position.

**Fig. 2:**
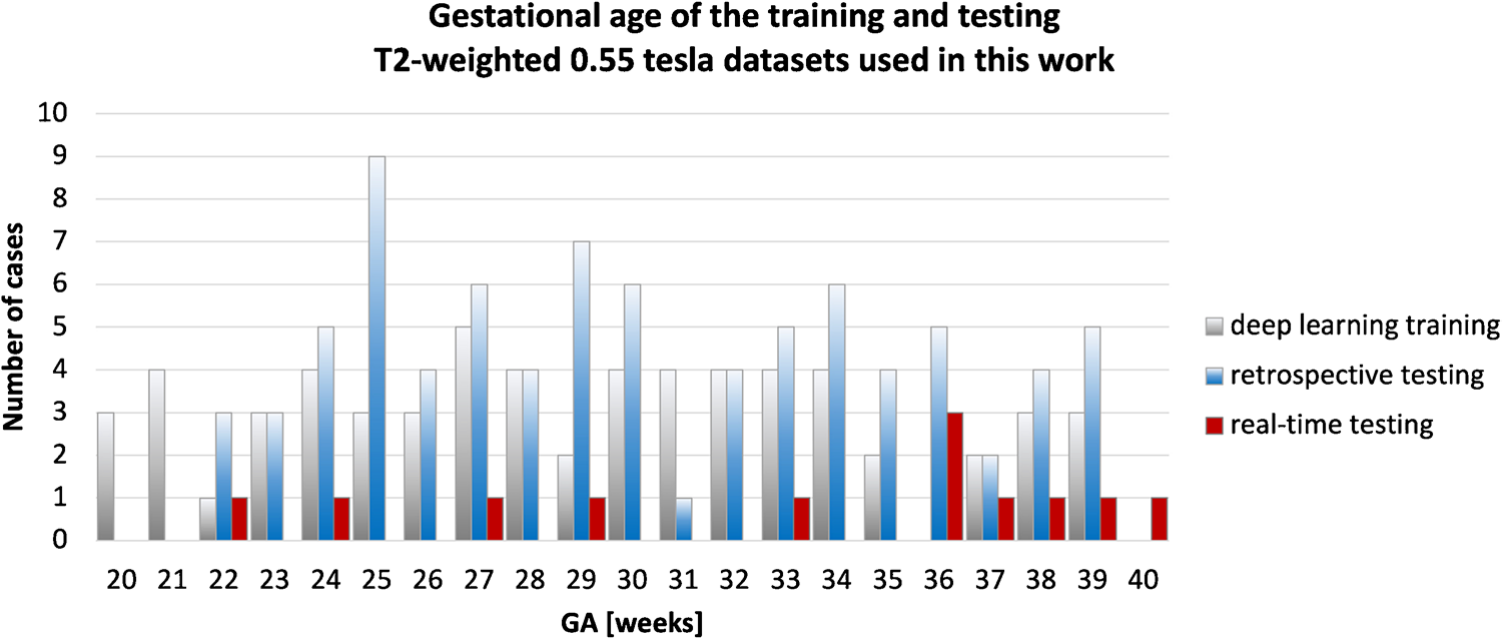
Gestational age distributions of the 0.55-T T2W datasets used in this work for the training of the deep learning models, retrospective image quality evaluation, and real-time testing. *GA*, gestational age

The 0.55-T datasets used in this work were acquired between 2022 and 2024 and cover 20–40 weeks gestational age (GA) range (Fig. 2) including:

- The deep learning training cohort: 384 stacks from 62 fetal datasets from 20–39 weeks GA range acquired during 05/2022–01/2023 period;
- The retrospective evaluation (quantitative and qualitative) cohort: 83 fetal datasets from 22–39 weeks GA range acquired during 02/2023–08/2023 period;
- The real-time testing cohort: 12 fetal datasets from 22–40 weeks GA range acquired during 01/2024–02/2024 period.

The main selection criteria for the retrospective testing cases were singleton pregnancy, > 22 weeks GA, no significant structural fetal pathologies, good in-plane image contrast with high SNR, clear visibility of the whole fetus, and no breaks during ssTSE acquisition. This is a heterogeneous cohort with the maternal BMI varying between 22–43, different placental and fetal findings, and varying volume of amniotic fluid. Out of 88 originally inspected retrospective evaluation datasets, only four (5%) were excluded due to suboptimal image quality in the majority of stacks with severe in-plane signal loss caused by shading, extreme motion artifacts, or other acquisition-related factors. These cases were excluded because there was not enough image information (i.e., good-quality slices) for T2W 3-D SVR reconstructions. Furthermore, we did not include cases below 22 weeks due to the small size of the fetal organs compared to the intrinsic large slice spacing and low resolution in 0.55-T datasets that would be expected to compromise output reconstruction quality. This work focused only on cases without extreme structural anomalies since they represent the primary cohort of the available research 0.55-T scans at our institution.

### Automated T2-weighted three-dimensional reconstruction

**Fig. 3:**
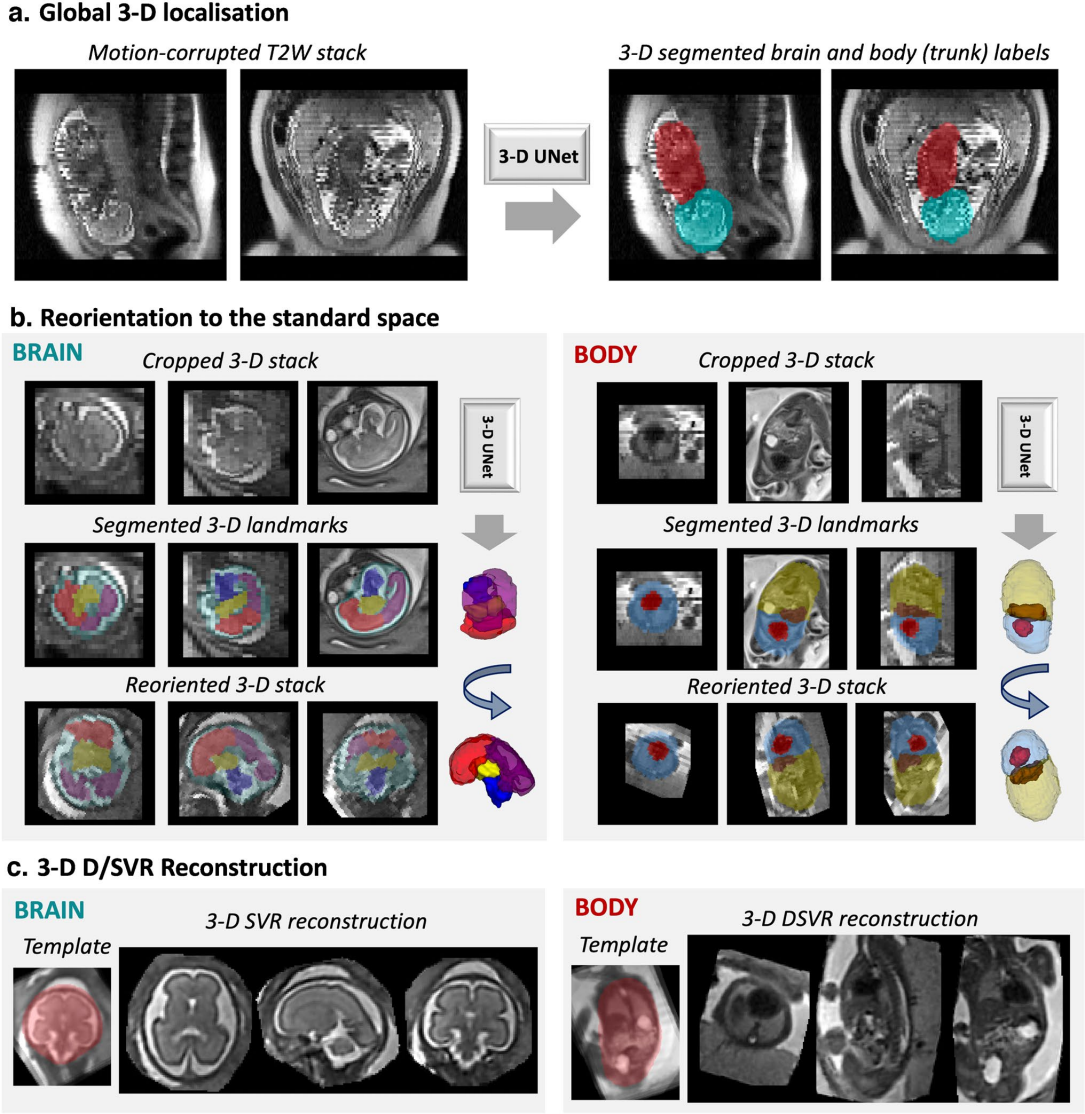
Proposed automated combined T2W 3-dimensional brain SVR and body DSVR reconstruction pipeline for T2W 0.55-T fetal MRI including global T2W 3-D localization in motion-corrupted stacks (**a**), T2W 3-dimensional reorientation to the standard space (**b**), and average template creation and D/SVR reconstruction (**c**). *3-D*, 3-dimensional; *DSVR*, deformable slice-to-volume reconstruction; *SVR*, slice-to-volume reconstruction; *T2W*, T2-weighted

The proposed automated pipeline for combined T2W 3-D brain + body D/SVR reconstruction is summarized in Fig. 3. This is an extension of the previous work for automated T2W 3-D reconstruction of the fetal brain and thorax [15, 31] with both brain and body regions of interest (ROIs) trained on 0.55-T datasets using medical open network for artificial intelligence (MONAI) [32] network implementations. It includes global T2W 3-D localization of the brain and trunk in all stacks, followed by landmark-based reorientation to the standard radiological space, template selection, and classical rigid SVR and DSVR reconstruction. An additional reorientation step is applied to the final reconstructed images for refined alignment.

Implementation of the global localization of the fetal head and body (Fig. 3) is based on a classical MONAI T2W 3-D UNet [33] segmentation network trained on 384 stacks from 62 0.55-T datasets with GA range 20–39 weeks with semi-manually created labels. Next, the reorientation of the brain and body ROIs to the same canonical space (Fig. 3) of all stacks is added in order to correct for large rotation and translation motion. It is implemented similarly to the previous works [15, 31] using MONAI T2W 3-D UNet of segmentation of four body (thorax, abdomen, heart, liver) and four brain (anterior brain, posterior brain, deep grey matter, cerebellum + brainstem) landmarks in ROI-cropped individual stacks. The networks were trained on 195 0.55-T cropped images with varying orientations and degrees of motion and semi-manual labels. The segmented landmarks are used for reorientation to the standard radiological space using classical point-based registration. After localization and reorientation, the processed stacks of slices are passed to the *stack-selection* [15] SVRTK function for generation of the average template space and mask and rejection of low-quality motion-corrupted stacks.

The final selected stacks are used in the rigid SVR [8] and rigid + deformable DSVR [9] functions for T2W 3-D brain and body reconstructions (Fig. 3) that were optimized to 0.55-T datasets with additional structure-based outlier rejection. Taking into account the native stack resolution (1.2 × 1.2 × 4.5 mm), the output resolution for T2W 3-D reconstructed images was selected as 1.0 mm.

### Implementation and evaluation details

All pipeline components (deep learning and C +  + reconstruction) are combined into one bash script. The code for the proposed brain and body reconstruction pipeline is publicly available at *auto-proc-svrtk* GitHub repository.^3^ The proposed 0.55-T T2W 3-D D/SVR reconstruction pipeline is publicly available as a standalone Docker application at SVRTK Docker repository^4^ that includes all required software installations as well as the network weights. The Docker is based on only central processing unit (CPU) processing and is executed on the CPU for straightforward deployment purposes. The recommended minimum Docker settings are > 20 gigabyte random-access memory and > height CPUs.

The evaluation of the proposed D/SVR pipeline is based on a set of retrospective datasets. The quantitative evaluation on 30 datasets is based on the localization distance and reorientation rotation errors and label Dice. The qualitative evaluation on 83 datasets is based on the grading of T2W 3-D reconstructed brain and body images in terms of image quality and visibility of organs by researchers with extensive experience in fetal MRI. The quality scores for the output T2W 3-D D/SVR images are as follows: 1—failed; 2—poor, 3—acceptable, 4—good (similar to the grading scheme in [5]). The evaluation was performed by three researchers with extensive experience in fetal and neonatal MRI (neuroradiologist: AL (2 years), clinical researcher: MH (> 5 years), technical researcher: AU (> 5 years)).

In addition, we run a supplementary comparison experiment to state-of-the-art reconstruction method based on implicit neural representation for slice-to-volume reconstruction (NeSVOR) [17] on five cases from 22 to 38 weeks GA range in order to demonstrate general performance in terms of reconstruction quality and time.

### Gadgetron-based scanner deployment of three-dimensional reconstruction pipeline

The entire online pipeline was implemented using Gadgetron. As described above in Fig. 3 step 1, the reconstructed stacks are exported to the dedicated GPU-equipped Gadgetron server immediately after acquisition, and D/SVR reconstruction is subsequently performed, triggered by a 5-s dummy sequence. The total reconstruction time is approximately 6:42 ± 3:13 min, and the results are available immediately on the scanner console.

After all ssTSE stacks are acquired, a 5-s “launch” sequence is run as part of the protocol (Fig. 3 step 2). This establishes a connection between the scanner and the server and launches the SVR Docker container on the server as a detached subprocess, allowing the acquisition and reconstruction of the subsequent sequences in the exam card. The acquired data from the launch sequence is discarded. The final reconstructions for the body and brain are saved in the Gadgetron server.

As a last step to bring the D/SVR reconstruction to the scanner host in a controlled manner, two 1-s “pull” sequences are run towards the end of the protocol. A modified, highly accelerated magnetization-prepared rapid gradient echo sequence is employed for this purpose with the matrix size matched to the expected reconstruction. All brain reconstructions are resampled to an image matrix of 128 × 128 × 128 (128 slices) and the body reconstructions to 256 × 256 × 256 (256 slices) prior to this step. Resampling ensures consistent dimensions for reconstructed volumes, preserving the field-of-view across sequences without compromising result quality (e.g., cropped structures or missing slices). Similar to the sequence used to launch the SVR processing, these sequences are designed to establish the connection to the external server. The acquired data is then overwritten with the T2W 3-D SVR volume matrices, injected into the vendor image reconstruction chain, accessed by the scanner host, and subsequently exported to PACS.

### Implementation details

The Gadgetron framework was installed on the dedicated external server and connected to the internal network of the MRI scanner. The sequences were modified to include the Gadgetron emitter and injector functors, enabling raw data to be sent to the external server for processing and the processed image data to be re-integrated into the scanner reconstruction pipeline. For each of the three tasks presented in this work, a pair of configuration files was created and the configuration file stored in the scanner host, defined by the sequence, points to the reconstruction and processing configuration file that is stored in the external server. The latter assembles the reconstruction and processing Gadgets the data streams through, including the Python-scripted external-language interface Gadgets that were developed for each task.

The code for the proposed Gadgetron-based D/SVR scanner integration for 0.55-T fetal MRI is publicly available at *gadgetron-svrtk-integration* GitHub repository.^5^

## Results

The proposed Gadgetron-deployed brain + body D/SVR reconstruction pipeline was evaluated on 83 retrospective 0.55-T datasets and on (in utero) prospective real-time testing of the integration step on 12 cases.

### Retrospective testing of three-dimensional reconstruction pipeline

The results of the quantitative evaluation of the global brain and body localization and reorientation on 30 datasets (with 60 stacks in total) within the 22–39 weeks GA range are presented in Table 1. The selected stacks have varying uterus and fetal acquisition planes. An evaluation was performed vs. manually created ground truth T2W 3-D labels. Similarly to [15], the T2W 3-D UNet showed robust performance for both brain and body with a relatively high Dice score and average 6.067 ± 1.950 (brain) and 7.836 ± 3.027 (body) mm center point distance errors for both early and late GA ranges. The proposed T2W 3-D localization network ensures continuity of the individual labels in T2W 3-D space that does not require additional post-processing in comparison to the conventional T2W 2-D slice-wise fetal brain nnUNet approach [11]. The quantitative evaluation of the reorientation of the brain and body to the standard radiological space was performed compared to the manually reoriented images. The landmark-based approach showed robust performance for both the brain and body with 11*.*947 ± 6*.*626 (brain) and 15*.*549 ± 12*.*487 (body) degree rotation error ranges which are acceptable ranges for a classical registration method. The higher rotation error for early GA datasets is due to the lower visibility of landmarks.

**Table 1:** Retrospective quantitative evaluation of global localization and reorientation to the standard space on 30 datasets with 60 stacks in terms of Dice score, localization distance error (mm), and rotation error (degrees)

Gestational age (weeks)	Dice score	Distance error (mm)	Rotation error (degree)
Brain ROI			
22–25	0.848 ± 0.046	5.819 ± 1.639	14.679 ± 6.009
26–31	0.879 ± 0.034	5.597 ± 1.702	10.706 ± 6.950
32–39	0.895 ± 0.026	6.783 ± 2.321	10.456 ± 6.331
Body ROI			
22–25	0.852 ± 0.028	6.569 ± 2.840	20.954 ± 13.166
26–31	0.840 ± 0.030	8.441 ± 2.617	15.140 ± 14.164
32–39	0.854 ± 0.031	8.498 ± 3.316	10.554 ± 7.286

*ROI*, region of interest

**Fig. 4:**
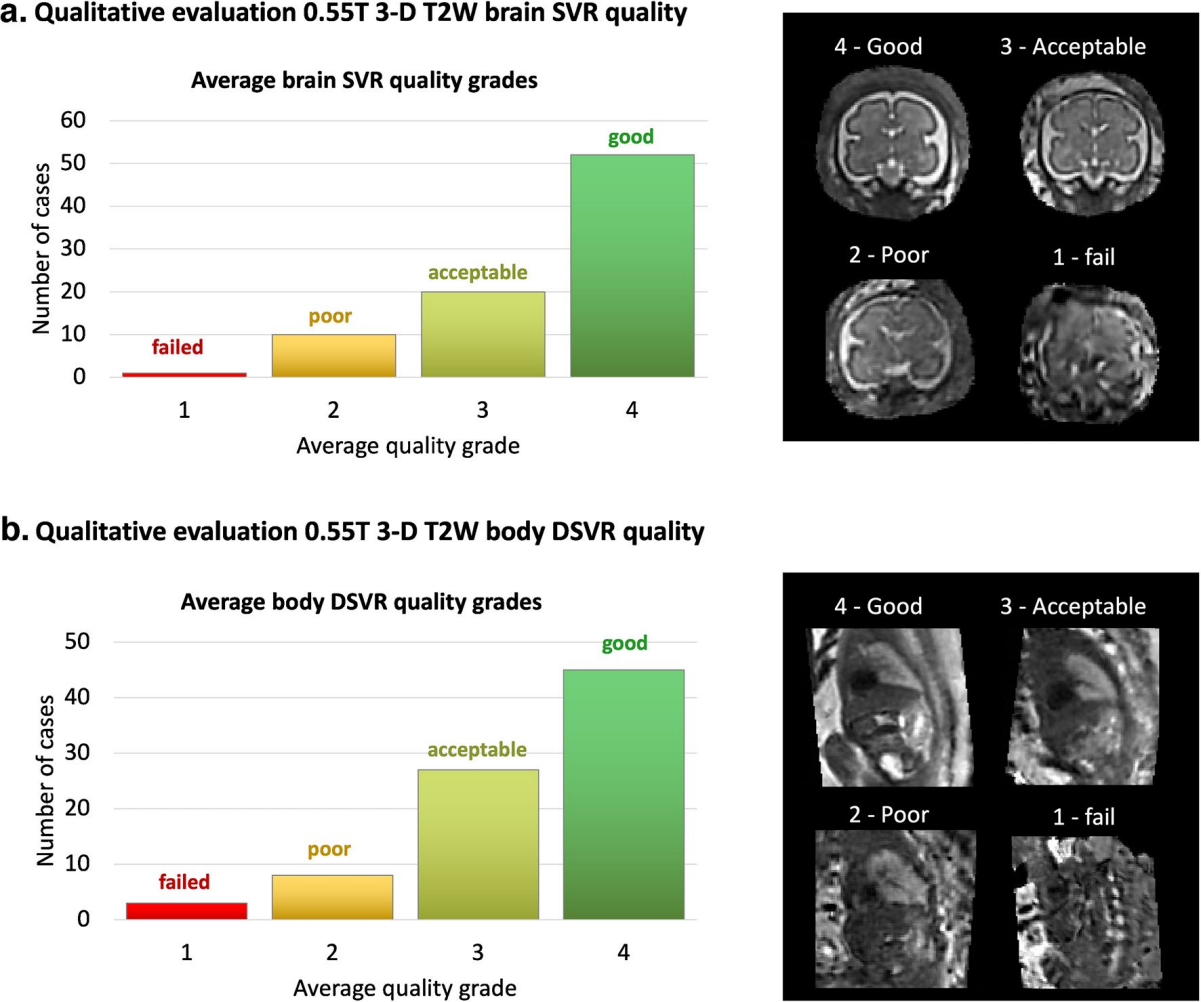
Retrospective qualitative evaluation of the 3-D T2-weighted brain (**a**) and body (**b**) reconstruction quality on 83 0.55-T datasets from 22–39 GA range. Grading scheme: *red* (1) = failed; *yellow* (2) = poor; *lime green* (3) = acceptable; *emerald green* (4) = good. *DSVR*, deformable slice-to-volume reconstruction; *GA*, gestational age; *SVR*, slice-to-volume reconstruction; *T2W*, T2-weighted; *T*, tesla

Qualitative evaluation of the automated D/SVR 0.55-T reconstruction results was performed on 83 datasets (not used in training) for both the brain and body based on defined quality scores. The results in Fig. 4 demonstrate that the pipeline has relatively stable performance with acceptable and good reconstruction quality for both the brain and body in the predominant (> 85%) proportion of the datasets. In brain reconstructions, 63% had good and 24% had acceptable quality. In body reconstructions, 54% had good and 33% had acceptable quality. The high proportion of acceptable quality cases was due to intrinsically suboptimal image signal quality specific to 0.55 T. The lower quality grades were in the early (due to small size) and late (due to the lower cortical and tissue organ contrasts) GA ranges and severe motion corruption cases with a high proportion of in-plane signal loss.

**Fig. 5:**
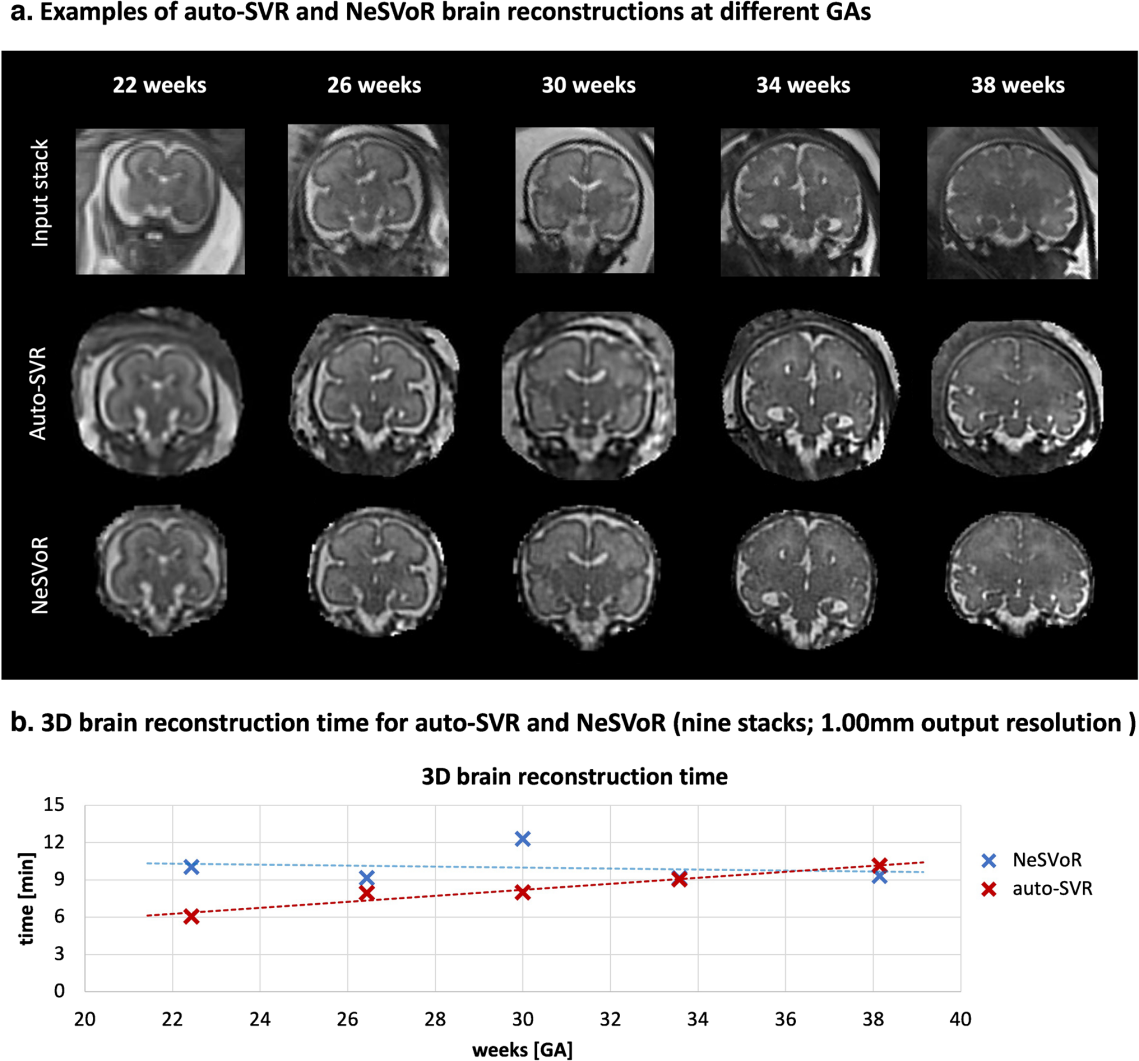
Examples of T2W 3-D proposed auto-SVRTK vs. NeSVoR [17] brain reconstructions (**a**) and total reconstruction time vs. GA (**b**). *auto-SVR*, automated slice-to-volume reconstruction; *GA*, gestational age; *NeSVoR*, implicit neural representation for slice-to-volume reconstruction

The main aims of this work are the general feasibility study for 0.55-T and scanner-based integration of classical T2W 3-D reconstruction and do not include a detailed comparison with a large number of the recently proposed novel SVR approaches, i.e., other methods can be interchanged and deployed on the scanner in a similar format. Furthermore, the recent two independent works [34, 35] from another research group already evaluated the performance of the proposed automated SVR pipeline on a set of multi-center 1.5-T and 3-T acquisition protocols and confirmed similar image quality vs. state-of-the-art GPU-based brain reconstruction NeSVoR method [17]. The result of an additional retrospective experiment illustrated in Fig. 5 comparing the proposed offline automated SVR and NeSVoR for five 22–38 weeks GA 0.55-T datasets also confirms these findings. The outputs of both methods have similar global features but the tissue signal texture in SVRTK-reconstructed images is smoother but less noisy than that in the NeSVoR results. This is potentially related to the differences in super-resolution reconstruction and regularization methods as well as the lower SNR and resolution of 0.55-T datasets. NeSVoR outputs cover the brain ROI with a tight mask but SVRTK allows reconstruction of the background ROI. The average reconstruction time per case (with nine stacks and 1.00-mm output resolution; default settings; on the same machine) was also within the similar range: 6–10 min (depending on GA) for automated SVR and 9–10 min for NeSVoR (Fig. 5).

### Gadgetron-based scanner deployment of three-dimensional reconstruction pipeline

**Fig. 6:**
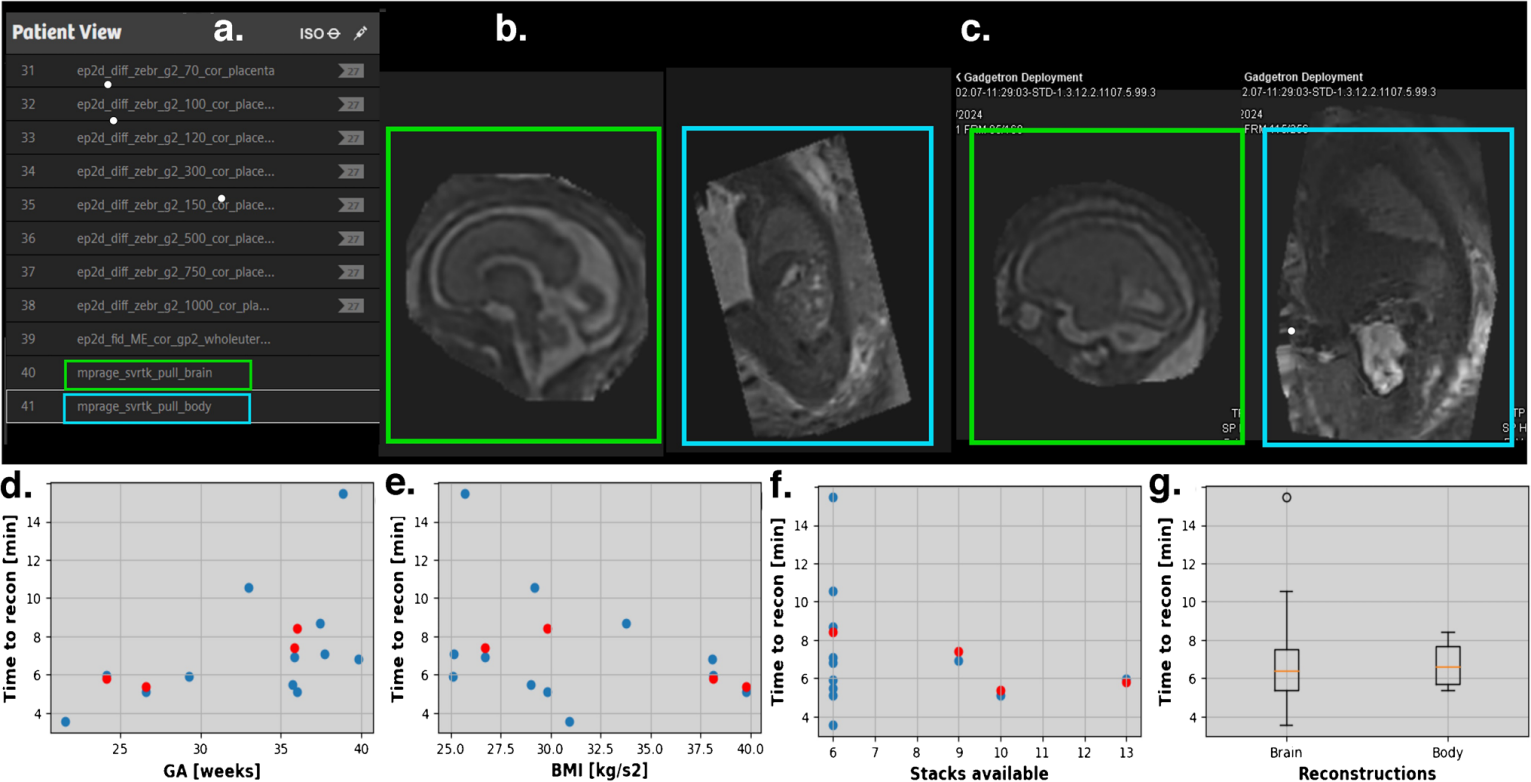
Results of the real-time deployment. **a** Screenshot of the end of the scanning protocol with the pull sequences. **b**, **c** Resulting brain (*green box*) and body (*blue box*) 3-D T2W reconstructions on the scanner console during the MR scan for two cases. **d**, **e**, **f** Quantitative assessment of the time required for the online reconstructions against GA at scan, BMI, and number of stacks. **g** Box plots illustrating the spread in time until the reconstruction was available online. *BMI*, body mass index; *GA*, gestational age

For the 12 prospective cases, T2W 3-D reconstructions were launched and made available on the scanner console during the fetal MRI scan, to view using all visualization options and to be archived with all acquired data at the end of the acquisition (Table 2). The output image quality was evaluated by two clinicians using the specified grading scheme (1 to 4). Of the 12 cases assessed, 10 were rated as acceptable to good, while two datasets (one from a healthy 38-week GA fetus and another from a 22-week GA fetus with T1DM) were deemed unsuccessful due to suboptimal original image quality. The overall median was 2. The launch and pull sequences on the protocol were moved freely by the radiographer performing the scan, with the launch sequence being acquired when a sufficient number of slices was obtained and the pull sequences performed after subsequent long diffusion sequences were acquired, at the end of the entire scan. Figure 6 illustrates such a setup with the corresponding reconstructions for the brain (green boxes) and body (blue boxes). The time between the launch and the availability of the reconstruction for the brain was, on average, 7.23 min (median 6.39 min, std = 3.03 min) and for the body 6.50 min (median 6.41 min, std = 1.27 min), depending on the number of stacks, subject GA, and computational system load. On average, no correlation of the processing time against BMI, GA, or the number of stacks was observed (see the graphs in Fig. 6).

**Table 2:** Demographics and results for the prospective cases

	GA (weeks)	BMI (kg/m^2^)	Brain (min)	Body (min)	Stacks
1. Healthy	37.71	25.17	07:05	-	6
2. Healthy	37.42	33.80	08:42	-	6
3. Healthy	35.7	29.0	05:29	-	6
4. T1DM	21.57	30.92	03:37	-	6
5. Healthy	33	29.2	10:36	-	6
6. IBD	29.3	25.1	04:52	-	6
7. Healthy	39.85	38.09	06:48	-	6
8. Healthy	38.85	25.71	15:28	-	6
9. Fibroids	35.85	26.73	06:54	07:26	9
10. CDH	26.57	39.79	05:07	05:25	10
11. Healthy	36	29.83	05:01	08:35	6
12. PPROM	24.14	38.16	05:55	05:56	13

Time refers to the time from the launch of the reconstruction on the scanner after the acquisition of the last T2W stack to the reconstruction being readily available on the scanner console computer

*BMI*, body mass index; *CDH*, congenital diaphragmatic hernia; *GA*, gestational age; *IBD*, inflammatory bowel disease; *PPROM*, preterm prolonged rupture of the membranes; *T1DM*, type 1 diabetes mellitus

## Discussion

In this work, we designed and implemented the first prototype pipeline for automated T2W 3-D D/SVR combined reconstruction of the fetal brain and body T2W at low-field MRI deployed on 0.55-T scanner via Gadgetron resulting in motion-corrected T2W 3-D reconstructions being available on the scanner console during the examination. While D/SVR has previously been demonstrated at 1.5 T and 3 T, this work extends its application to low-field fetal MRI scans. Low-field scans pose unique challenges, such as lower SNR and reduced resolution. Thus, the availability of D/SVR at low-field strengths represents a significant step towards making high-quality fetal MRI more accessible for wider research and clinical use.

The main novel components are hence the compilation of fetal brain + body D/SVR methods into one combined pipeline, the application of T2W 3-D image-domain reconstruction methods to low-field fetal MRI, and the integration of these methods into the scanner environment.

This workflow not only allows D/SVR to be run for all patients scanned in the low-field scanner automatically and immediately, with results stored in PACS alongside the acquired images, but it also reduces the workload on radiographers and researchers who run the processing pipeline offline post-scan.

Quantitative and qualitative evaluation of the proposed automated D/SVR pipeline optimized for T2W 0.55-T data on 83 retrospective cases demonstrated general suitability of using T2W 3-D reconstruction methods for low-field MRI. It also showed comparable performance with the most recent state-of-the-art deep learning method for the fetal brain [17] in terms of both reconstruction quality and time. This suggests that the majority of the currently available SVR methods are generally interchangeable and that the SVR reconstruction quality is primarily defined by the input MRI data.

This prototype automated pipeline also allowed integration of D/SVR methods directly into the scanner environment enabling T2W 3-D reconstructions to be visualized on the scanner console during the examination. The results of real-time in utero prospective testing on 12 cases confirmed the robustness of the method, no interference with the scan acquisition, and the ability to achieve good data quality. The T2W 3-D brain reconstruction from six stacks was available on the console within 7 min on average, and only one case took > 10 min, which was attributed to a temporary performance slowdown of the console computer. One of the major advantages of the proposed real-time deployment solution is that running T2W 3-D SVR reconstructions directly during scanning allows straightforward import of T2W 3-D images into PACS (or similar systems) together with raw stack data without any extra offline processing steps after acquisitions.

The reconstructed image quality at 0.55 T is inherently lower, as anticipated, compared to conventional 1.5-T and 3-T results. This difference is primarily due to the reduced sharpness of features and tissue interfaces associated with the lower field strength, reflecting the expected limitations in the quality of the original stack data. Concurrently, recent studies by [34, 35] independently evaluated the proposed automated SVR pipeline for fetal brain reconstructions using a set of multi-center 1.5-T and 3-T acquisition protocols, comparing its performance against alternative reconstruction pipelines [11, 17]. Their findings affirmed the feasibility of applying the current pipeline to higher field strengths, demonstrating that the proposed auto-SVR method delivers superior image quality.

Since the primary aims of this work are the general D/SVR feasibility assessment for 0.55-T and real-time integration, we did not include an assessment of the pipeline performance on the extreme motion and signal loss datasets. In clinical practice, these cases normally represent only a small proportion of datasets and should potentially be addressed by real-time acquisition quality control since the lack of original T2W 2-D slice image information will make any T2W 3-D reconstructed images unreliable by definition.

Our future work will also focus on detailed technical formalization of the minimum input image requirements and an automated real-time stack quality classification module that could also provide active guidance for the re-acquisition of motion-corrupted stacks [20].

In terms of improvement of performance of the pipeline for early GA cases, further optimization will be required based on further retraining of the localization and reorientation networks on early GA cases and optimization of the template generation and slice-to-volume registration modules. Furthermore, the proposed auto-D/SVR pipeline was trained and tested only on datasets without major structural anomalies. However, integration into clinical practice will require it to be operational for a variety of clinical conditions. As the next step, we will adapt the pipeline for the cases with extreme structural deviations in anatomy such as severe ventriculomegaly, spina bifida, and congenital diaphragmatic hernia, as well as twin pregnancies. This will be achieved by further retraining of the networks on a wider range of atypical cases and additional classification and preprocessing steps.

One of the potential limitations of this work is the reliance on the classical D/SVR methods for the reconstruction part and the expected sensitivity of the landmark reorientation method to low-quality stacks. Even though the full pipeline is operational, integration of more advanced deep learning super-resolution reconstruction and pose estimation methods [17] would be beneficial for robust performance for severe motion corruption cases. This will be addressed in the future work based on a thorough investigation of the optimal D/SVR reconstruction pipeline modules for structural MRI along with the extension with automated organ parcellation [36] for volumetry reporting during scanning. Furthermore, we will explore solutions for deep learning image recovery specific for datasets with severe signal and contrast loss.

The fact that the proposed structure of the Gadgetron-based scanner deployment relies on the external launch of the D/SVR docker application can also be considered a form of limitation since the activation and the transfer of results back to the scanner rely on the file availability logic. One of the possible solutions could be based on more advanced integration of the reconstruction code into the Gadgetron interface, e.g., dynamic masking, reorientation [18, 19], and sequential addition of stacks to the reconstruction function.

Another real-time implementation limitation is the requirement for sufficient time in the sequence list following the trigger of the SVR for the reconstruction process to be completed and the results retrieved in the scanner within the scan duration, which can be limiting for different protocol setups. Currently, in case the scan is completed and the results are not yet available, they can be retrieved post-scan by running the pull sequence at any time. However, a more time-efficient iterative approach will be explored in the future. It involves initializing the online SVR pipeline as soon as the first stack is acquired, with the subsequent stacks incrementally added to the reconstruction immediately upon acquisition, thus accelerating the overall process.

While implemented here for a 0.55-T system from one vendor, generalization of the online deployment pipeline for other systems operating at 1.5–3-T field strengths from a wider range of vendors is a logical next step, supported by the use of open-source tools at every step other than the MR sequence itself.

Another essential aspect is the qualitative assessment of T2W 3-D D/SVR images at 0.55 T in terms of added diagnostic value and image quality limitations. In general, T2W 3-D reconstructions improve information content in terms of providing continuous information in T2W 3-D space which is beneficial for both interpretation of T2W images and biometry or volumetry measurements. However, a further thorough wider clinical evaluation is required in order to assess the relevance and reliability of T2W 3-D 0.55-T images for clinical practice along with a comparison to 1.5-T and 3-T data.

We will also introduce a SVR-specific watermark label in the background of T2W 3-D reconstructed images in order to mark them as processing outputs and to distinguish them from the raw scan data.

## Conclusion

This study shows the feasibility of a real-time scanner-integrated solution for 0.55-T fetal brain and body T2W 3-D reconstruction. The initial step towards integration of T2W 3-D D/SVR reconstruction methods directly into the clinical environment has been developed and has the potential to enable straightforward processing and reporting of fetal MRI scans. Furthermore, this is the first reported work on T2W 3-D reconstruction for low-field fetal MRI. Our future work will focus on further optimization and integration of the D/SVR reconstruction and image quality control into the scanner environment for real-time processing for higher field strengths.

## Data Availability

The fetal MRI data used in this work were acquired as part of the ethically approved research studies: MEERKAT (REC: 21/LO/0742), MiBirth (REC: 23/LO/0685), and NANO (REC: 22/YH/0210) in accordance with the ethical standards as laid down in the 1964 Declaration of Helsinki and its later amendments. These studies have research ethics committee (REC) approval by the Health Research Authority boards of the following: London (including -Fulham, -South East, -Riverside, Dulwich, -West London and GTAC, and -Brent) and South East Scotland. Contact address: Health Research Authority, 2 Redman Place, Stratford, London, E20 1JQ. The individual fetal MRI datasets used for this study are not publicly available due to ethics regulations. For more information please contact Jana Hutter jana.hutter@kcl.ac.uk.

## Footnotes

1 Auto-SVRTK toolbox repository: https://github.com/SVRTK/auto-proc-svrtk

2 Gadgetron repository: https://github.com/gadgetron/gadgetron

3 Fetal MRI analysis *auto-proc-svrtk* repository: https://github.com/SVRTK/auto-proc-svrtk

4 SVRTK auto D/SVR reconstruction docker: https://hub.docker.com/r/fetalsvrtk/svrtk; tag *general_auto_amd.*

5 Gadgetron *SVRTK* integration repository: https://github.com/SVRTK/gadgetron-svrtk-integration

## Declarations

All experiments were performed in accordance with relevant guidelines and regulations. Informed written consent was obtained from all participants.
